# The Mediating Role of Vaccine Hesitancy in Influenza Vaccination Uptake and Intention Among Older Adults in Urban China: Based on a Structural Equation Modeling Study

**DOI:** 10.3390/vaccines13121249

**Published:** 2025-12-16

**Authors:** Shuai Yuan, Yuxing Wang, Yuanruo Xie, Jianing Dai, Sean X. Leng, Lili You

**Affiliations:** 1 Department of Health Policy and Management, Bloomberg School of Public Health, Johns Hopkins University, Baltimore, MD 21205, USA; shuai.yuan@jhu.edu; 2 Institute for Hospital Management, Tsinghua University, Shenzhen 518000, China; 3 School of Health Policy and Management, Chinese Academy of Medical Sciences and Peking Union Medical College, Beijing 100080, China; wangyx@student.pumc.edu.cn (Y.W.); daijn@student.pumc.edu.cn (J.D.); 4 Department of Health Policy and Management, Mailman School of Public Health, Columbia University, New York, NY 10032, USA; yx3031@cumc.columbia.edu; 5 Division of Geriatric Medicine, Department of Medicine, Johns Hopkins Bayview Medical Center, Baltimore, MD 21224, USA

**Keywords:** vaccine hesitancy, influenza vaccination, influenza intention, structural equation modeling, SEM, older adult

## Abstract

**Background**: Influenza presents significant risks to older adults; however, vaccination coverage in China remains low despite robust recommendations. Factors such as vaccine hesitancy, physician recommendations, health status, and socioeconomic conditions influence vaccination rates. This study uses large-scale influenza vaccination data from urban older adults in six cities and applies structural equation modeling to investigate the determinants of both influenza vaccination uptake and future intention. **Methods**: A cross-sectional survey was conducted from December 2024 to January 2025 across six major Chinese cities, involving 13,363 community-dwelling adults aged ≥60 years. Vaccine hesitancy was measured using the validated 5C scale. Structural equation modeling with weighted least squares mean and variance adjusted estimation was employed to assess direct and indirect effects of physician recommendation, socioeconomic status, medical status, and subjective health on influenza vaccination uptake. **Results**: The vaccination uptake rate is 34.05%, while the intention rate is 32.20%. Vaccine hesitancy is the strongest negative predictor of vaccination (β = −0.488, *p* < 0.001). Physician recommendation has the largest total effect (β = 0.351), with 45.60% of this effect mediated through reduced vaccine hesitancy. Medical status is directly associated with lower uptake; it consistently promoted intention. Higher socioeconomic status also positively affected vaccination (total effect = 0.167), partly via lower hesitancy. **Conclusions**: Vaccine hesitancy serves as a pivotal mediator in influenza vaccination uptake and intention among Chinese older adults. Strengthening physician recommendations and addressing socioeconomic disparities are key strategies to reduce hesitancy and improve coverage.

## 1. Introduction

Influenza is a pervasive viral respiratory disease that imposes a heavy burden on global public health. According to the World Health Organization (WHO) and the Chinese Center for Disease Control and Prevention (China CDC), high-risk groups include infants, young children, pregnant women, healthcare workers, individuals with chronic medical conditions, and, most notably, older adults [1,2,3,4,5]. Annual vaccination is strongly recommended for high-risk groups, as it can mitigate the impact of various life-threatening illnesses [6,7,8,9]. China’s rapidly aging population presents a substantial public health challenge. By the end of 2024, individuals aged 60 and older are projected to comprise 22.0% of the national population [10]. The health of older adults has emerged as a critical concern for both the government and society. In China, estimated influenza vaccination rates for adults aged >60 years across the 2019–2023 seasons were 1.57%, 3.03%, 3.75% and 4.16%, respectively [11]. Currently, influenza vaccine is generally voluntary and paid for out-of-pocket, rather than being included in the mandatory National Immunization Program [12]. Some cities, such as Beijing, provide free influenza vaccines to registered older adults, whereas, in many other cities, the cost remains a barrier [13]. In this context, promoting preventive health behaviors among older adults, particularly vaccination, has become a strategic priority for public health policy and initiatives aimed at healthy aging. Despite strong recommendations from national and international health authorities, influenza vaccine uptake among older adults in China remains suboptimal, underscoring the need to understand the underlying behavioral and psychological determinants [2,14]. This study specifically focuses on urban older adults, as urban areas are characterized by high population density, which facilitates the rapid transmission of respiratory viruses [2].

Factors affecting vaccination include host-related characteristics, sociocultural influences, and socioeconomic status, and vaccine hesitancy, which is considered an important factor affecting vaccination rates [5,15,16,17]. In recent years, vaccine hesitancy- characterized as a delay in acceptance or outright refusal of vaccines despite their availability—has emerged as a critical psychological barrier [18,19,20]. From a public health perspective, vaccine hesitancy undermines herd immunity, heightens the risk of outbreaks, contributes to increased morbidity and mortality, and places additional strain on healthcare systems due to rising hospitalizations and associated costs [21]. The 5C scale is commonly employed to assess vaccine hesitancy and encompasses five dimensions: confidence, complacency, collective responsibility, calculation, and convenience [22,23]. Confidence refers to the extent to which an individual trusts the effectiveness and safety of vaccines, as well as the health care system [19]. Complacency reflects an individual’s perception of disease risk; when the perceived threat of a disease is low, individuals may feel less compelled to vaccinate. Constraints denote structural or situational barriers that hinder vaccination, including factors such as time, cost, accessibility, and convenience [23]. Calculation involves the propensity of individuals to actively seek and evaluate information regarding vaccines, including the pros and cons and the risk-benefit ratio; excessive calculation may result in decision delays [21]. Collective responsibility refers to the belief that individuals have a duty to protect others through vaccination. These factors are often interconnected. Socioeconomic status may influence an individual’s health literacy and trust in the healthcare system, and a lack of trust leading to vaccine hesitancy may be shaped by low socioeconomic status or limited education.

Furthermore, vaccination intention is a critical immediate antecedent to behavior. However, a discrepancy known as the “intention-behavior gap” often exists, where individuals intend to vaccinate but fail to do so due to various barriers. Therefore, examining both intention and actual uptake provides a more comprehensive understanding of the decision-making process.

Most current cross-sectional surveys on influenza vaccination in China are concentrated in a single city, with few studies conducted across multiple cities [16,24]. To address these gaps, this study draws on a large-scale, multi-city survey of older adults across six geographically and economically diverse Chinese cities: Beijing, Chengdu, Hangzhou, Qingdao, Shenzhen, and Chongqing. By employing structural equation modeling (SEM), we aim to (1) assess the direct associations between physician recommendation, socioeconomic status, medical history, subjective health, and both influenza vaccination uptake and intention; and (2) quantify the mediating effect of vaccine hesitancy in the relationships between these predictors and these outcomes, and identify pathway differences.

## 2. Materials and Methods

### 2.1. Setting and Participants

This cross-sectional study was conducted from December 2024 to January 2025 in six major Chinese cities—Beijing, Hangzhou, Qingdao, Shenzhen, Chongqing, and Chengdu. Community Health Service Centers (CHSCs) in China serve as primary healthcare hubs for urban residents, providing basic medical care, chronic disease management, and vaccination services [25]. In each city, 5—8 CHSCs were randomly selected using simple random sampling from the official list of all local CHSCs. Each CHSC was assigned a recruitment target of at least 300 participants.

Inclusion criteria were: (1) age ≥ 60 years; (2) ability to communicate effectively with interviewers; (3) permanent residence in the study for >6 months. Exclusion criteria were: (1) cognitive impairment or diagnosis of dementia and (2) serve acute illness preventing participation. Trained interviewers approached eligible individuals consecutively in waiting areas and invited them to participate. All interviews were conducted face-to-face, and questionnaires were configured to require completion of all items before submission, minimizing missing data.

A total of 13,754 individuals were invited to participate, and 13,363 completed questionnaires were retained after rigorous data cleaning. Invalid responses—including those from individuals younger than 60 years or with contradictory answers—were excluded to ensure data validity. The final response validity rate was 97.2%.

#### Sample Size Calculation

The sample size was calculated based on the 2023 influenza vaccination coverage rate of 4.16% among Chinese adults aged ≥60 years, as reported by the China CDC. We aimed for a high precision level with a margin of error (*δ*) of 0.45% and a 95% confidence level (*Z* = 1.96). The minimum required sample size was 8850. Accounting for an estimated non-response or inefficiency rate of 35%, we targeted a total sample of 11,948 participants across the six cities. Using the standard formula for cross-sectional studies:

$$n=\frac{Z_{1-\alpha /2}^{2}\times p(1-p)}{\delta^{2}}$$

### 2.2. Measures

This study aimed to test a theoretical framework in which vaccine hesitancy mediates the relationships between socioeconomic status and subjective health, medical history and two distinct outcomes: vaccination uptake and vaccination intention. The primary outcome variables were binary indicators of whether the participant had ever received the influenza vaccine during the past one year. Participants were asked: “Did you receive the influenza vaccine in the past 12 months?” (Yes = 1, No = 0). To assess future willingness, vaccination intention was measured as a secondary outcome. Participants were asked: “Do you intend to receive the influenza vaccine in the coming 12 months?” (Yes = 1, No = 0).

Vaccine hesitancy, the mediator, was assessed using the validated 15-item 5C scale, which captures five psychological antecedents of vaccination behavior: confidence, collective responsibility, complacency, constraints, and calculation. Responses were recorded on a 5-point Likert scale (1 = Strongly Disagree to 5 = Strongly Agree). Following standard scoring procedures, items from the confidence and collective responsibility subscales were reverse-coded so that higher total scores reflected greater vaccine hesitancy. The 5C scale served as the latent mediator in the SEM, representing the psychological barrier.

Four key predictors were included as exogenous variables: Physician recommendation was measured dichotomously (“Has a family physician ever recommended that you receive the influenza vaccination?”; Yes = 1, No = 0). Medical status was assessed via a checklist of chronic conditions, including hypertension, diabetes, hyperlipidemia, cerebrovascular diseases, and other cardiovascular diseases besides hypertension. Subjective health is defined as participants’ overall self-assessment of their health status and self-care capacity, represented by a continuous self-rated health score that ranges from 1 (Very Poor) to 5 (Very Good). In contrast, self-care ability is measured dichotomously, with responses indicating whether individuals possess full self-care capability (Yes = 1, No = 0). Self-rated health reflects global health perception, while self-care capacity reflects functional autonomy; together they capture multidimensional aspects of subjective health in older adults. Socioeconomic status was a composite indicator comprising educational level and monthly income. Educational level was categorized based on the Chinese system: primary school or below (≤6 years of schooling), junior high school (7–9 years), senior high school (10–12 years), and college or above (>12 years). monthly income was categorized as ≤2500 CNY, 2501–5000 CNY, 5001–7500 CNY, >7500 CNY. All variables were integrated into a path-analytic SEM to simultaneously estimate direct and indirect (via vaccine hesitancy) effects on influenza vaccination uptake and intention.

### 2.3. Statistical Analysis

All analyses were performed using R version 4.4.3. A structural equation model was specified with vaccination uptake as the endogenous variables, and vaccine hesitancy, subjective health, medical history, and physician recommendation as external variables. And we used the weighted least squares mean and variance adjusted (WLSMV) estimator, which is robust for categorical and non-normal data [26,27]. A *p*-value < 0.05 was considered statistically significant for path coefficients. The comparative fit index (CFI), Tucker–Lewis index (TLI), root mean square error of approximation (RMSEA), standardized root mean square residual (SRMR), and parsimony-normed fit index (PNFI) were used to evaluate the model.

## 3. Results

### 3.1. Characteristics of Participants

The survey includes 13,363 urban older adults in six Chinese cities—Beijing (16.31%, *n* = 2180), Chengdu (15.41%, *n* = 2059), Hangzhou (16.52%, *n* = 2208), Qingdao (16.01%, *n* = 2140), Shenzhen (16.86%, *n* = 2253), and Chongqing (18.88%, *n* = 2523). The sample shows that 7315 are female (54.74%) and 6048 are male (45.26%). Participants have a mean age of 69.52 years. The overall influenza vaccination uptake rate is 34.05% (*n* = 4550) and overall influenza intention rate is 32.20% (*n* = 4311).

### 3.2. Confirmatory Factor Analysis of Latent Constructs

Confirmatory factor analysis (CFA) is performed to validate the measurement model for four latent variables: vaccine hesitancy, medical history, socioeconomic status, and subjective health. All standardized factor loadings are statistically significant (*p* < 0.001), indicating robust relationships between the observed indicators and their respective latent constructs (Table 1). For vaccine hesitancy, measured by five items from the 5C scale, standardized loadings ranged from 0.408 (calculation) to 0.769 (confidence), with all items surpassing the commonly accepted threshold of 0.400, thereby suggesting adequate convergent validity. Reverse-coded confidence exhibited the highest standardized factor loading, followed closely by reverse-coded collective responsibility, while calculation showed the weakest contribution. The medical history latent variable, which comprises six chronic conditions, shows loadings between 0.477 and 0.555, reflecting consistent but moderate associations typical of multi-morbidity indicators. Socioeconomic status, constructed from education level and monthly income, demonstrated high loadings of 0.827 and 0.852, respectively, indicating that these indicators effectively captured the underlying socioeconomic status construct. Similarly, subjective health, assessed through two self-rated items, yields loading of 0.689 and 0.519, supporting its validity as a coherent latent dimension. Overall, the CFA results confirm that the measurement model is well-specified and appropriate for inclusion in the subsequent structural equation model.

### 3.3. Fitness for the SEM

Table 2 shows the model fit indices for both the vaccination uptake and vaccination intention models. The χ^2^/df ratio is 47.686. While this exceeds the traditional threshold of 5.000, it is a well-known artifact of the large sample sizes. Other key indices (CFI, TLI, RMSEA, SRMR, PNFI) fell within acceptable ranges, indicating a satisfactory model fit and suggesting that the structural equation model has a good over effect. Collectively, these fit indices support the adequacy of the hypothesized model.

### 3.4. SEM Results for Vaccination Uptake

Table 3 presents the SEM path coefficient for vaccination uptake. For the influenza vaccination uptake, vaccine hesitancy (*p* < 0.001, β = −0.488), subjective health (*p* = 0.003, β = −0.234), medical history (*p* = 0.036, β = −0.152) show significant negative effects on vaccination uptake. Physician recommendation (*p* < 0.001, β = 0.191) and higher socioeconomic status (*p* < 0.001, β = 0.125) show significant positive effects on vaccination uptake. For vaccine hesitancy, subjective health (*p* < 0.001, β = −0.447), medical history (*p* < 0.001, β = −0.334), physician recommendation (*p* < 0.001, β = −0.327), lower socioeconomic status (*p* = 0.013, β = −0.087) show significant negative effects.

**Figure 1:**
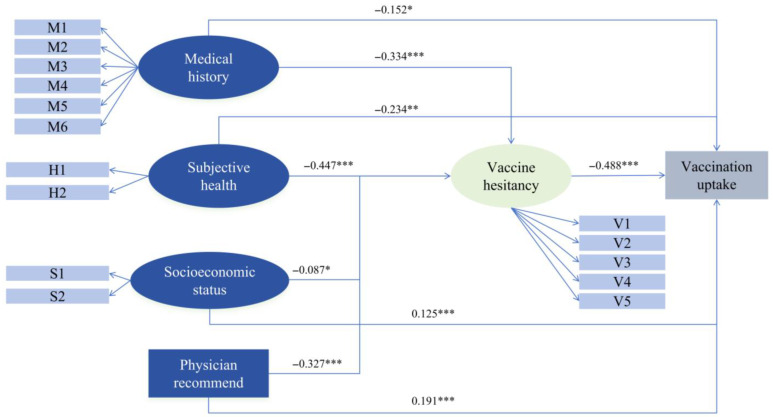
A path diagram of vaccination uptake using a structural equation model. Notes: M1 to M6 included hypertension, diabetes, hyperlipidemia, cerebrovascular diseases, other cardiovascular diseases apart from hypertension, and kidney disease; H1 represents health status, while H2 denotes self-care capacity; S1 corresponds to educational level, and S2 to monthly income; V1 to V5 encompass confidence, constraint, calculation, complacency, and collective responsibility; *** *p* < 0.001, ** *p* < 0.01, * *p* < 0.5.

Table 4 presents the decomposition of total effects into direct and indirect components. Physician recommend demonstrates the strongest total effect (β = 0.351), with both a significant direct effect (β = 0.191), and a substantial indirect effect mediated through reduced vaccine hesitancy (β = 0.160), indicating that 45.6% of its total effect operates via lowering hesitancy. Higher socioeconomic status is positively associated with vaccination uptake, with a direct effect of β = 0.125 and an indirect effect of β = 0.042 through decreased vaccine hesitancy, yielding a total effect of β = 0.167. Both medical status and subjective health exhibit suppression effects. Higher medical burden was directly associated with lower vaccination uptake (β = –0.152) but indirectly promotes vaccination by reducing vaccine hesitancy (β = 0.163), resulting in a near-zero total effect. Similarly, better subjective health directly discouraged vaccination (β = –0.234) but indirectly facilitates it through lower hesitancy (β = 0.218). These patterns suggest that vaccine hesitancy masks the true complexity of health-related predictors, highlighting the importance of modeling mediating pathways rather than relying solely on total effects. Figure 1 visually summarizes the SEM.

### 3.5. SEM Results for Vaccination Intention

Table 5 presents the SEM path coefficients for vaccination intention. For the vaccination intention, vaccine hesitancy (*p* < 0.001, β = −0.605) shows a significant negative effect. Medical status (*p* = 0.003, β = 0.197) and physician recommendation (*p* < 0.001, β = 0.129) show significant positive effects on vaccination intention. For vaccine hesitancy, subjective health (*p* < 0.001, β = −0.453), medical history (*p* < 0.001, β = −0.340), physician recommendation (*p* < 0.001,β = −0.325), and higher socioeconomic status (*p* = 0.020, β = −0.083) show significant negative effects.

**Figure 2:**
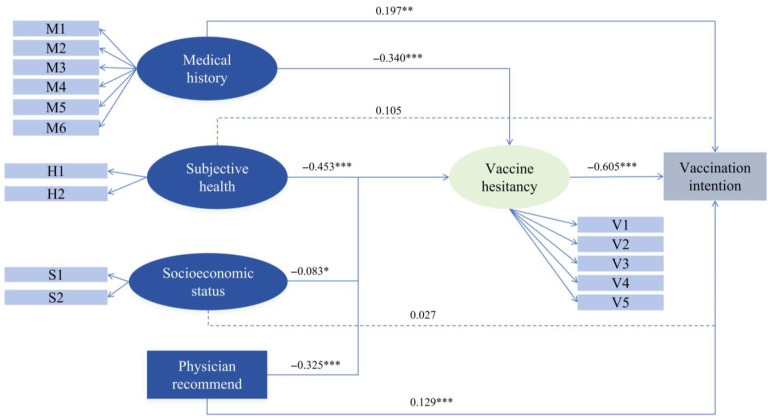
A path diagram of vaccination intention using a structural equation model. Notes: M1 to M6 included hypertension, diabetes, hyperlipidemia, cerebrovascular diseases, other cardiovascular diseases apart from hypertension, and kidney disease; H1 represents health status, while H2 denotes self-care capacity; S1 corresponds to educational level, and S2 to monthly income; V1 to V5 encompass confidence, constraint, calculation, complacency, and collective responsibility; *** *p* < 0.001, ** *p* < 0.01, * *p* < 0.5.

Table 6 presents the decomposition of total effects into direct and indirect components regarding vaccination intention. Medical status demonstrates the strongest total effect (β = 0.403). Subjective health and Socioeconomic status influenced vaccination intention almost exclusively through the indirect pathway, as their direct effects are non-significant. Physician recommendation influenced intention through both direct (β = 0.129) and indirect (β = 0.197), yielding a robust total effect (β = 0.326). Figure 2 visually summarizes the SEM.

## 4. Discussion

This study aimed to identify the key factors influencing both influenza vaccination behavior and intention among older adults in China, focusing specifically on the direct and mediating pathways involving physician recommendations, health perceptions, and socioeconomic status. The SEM shows a good fit, thereby supporting the validity of the theoretical framework and its effectiveness in elucidating the mechanisms underlying vaccination behavior and intention in this population.

Our analysis clearly identified vaccine hesitancy as a significant negative predictor of vaccination behavior, emphasizing its role as a primary barrier. This finding is consistent with previous research [28]. The factor analysis reveals that, among the 5C dimensions, confidence is the strongest indicator of the vaccine hesitancy construct. This means that vaccine hesitancy in Chinese older adults is fundamentally driven by a lack of confidence in vaccines. Calculation is the weakest indicator, suggesting that for Chinese urban older adults, the main barrier is not detailed risk-benefit calculation, but rather the fundamental trust in the vaccine. Therefore, intervention strategies should focus on trust-building, including the dissemination of scientifically accurate information, the active involvement of trusted healthcare providers in the recommendation process, and the implementation of peer education initiatives [29,30].

Vaccine hesitancy also functions as a significant mediator underscores its central role in the vaccination promotion ecosystem and indicating that interventions aimed directly at addressing hesitancy are likely to produce considerable improvements in coverage. Our study shows that medical history, subjective health, socioeconomic factors, and physician recommendations are statistically significant predictors of vaccine hesitancy, aligning with findings from previous research. Historically, chronic diseases were often viewed as contraindications to vaccination, which resulted in delays in vaccinating high-risk populations [31,32]. However, our findings indicate that an increase in chronic diseases correlates with a decreased risk of vaccine hesitancy. This trend likely stems from the fact that older individuals with multiple chronic conditions tend to have diminished confidence in their health and, as confirmed by our vaccination intention model, exhibit a significantly higher willingness to receive vaccinations for self-protection. This association may be linked to the crystallized health literacy of older people, due to their familiarity with diseases, medications and flu vaccines. As understanding of vaccines becomes more comprehensive and scientifically grounded, the contraindications associated with vaccination are clarified, leading to a correction in the general perception of chronic diseases as contraindications.

Our findings also contextualize the substantial impact of physician recommendations. Physician advice is the strongest positive predictor of uptake, with nearly half of this effect mediated by a reduction in vaccine hesitancy. In the information age, older adults are particularly susceptible to misperceptions and negative opinions [33,34]. Physicians, as professionals, can play a crucial role in addressing these misunderstandings [35]. This study shows that physician recommendations not only significantly increased the likelihood of vaccination but also diminished vaccine hesitancy among older adults. This underscores the role of physicians not only as sources of information but also as vital agents in building trust with patients [36,37]. These results corroborate previous studies that emphasize provider recommendations as a cornerstone of vaccine confidence and strongly support the World Health Organization’s advocacy for leveraging healthcare workers to combat hesitancy [35]. Consequently, interventions should prioritize trust-building strategies—such as transparent communication from family doctors—over complex information dissemination. Reinforcing provider recommendations thus serves as a critical mechanism to restore trust and improve vaccination uptake among older adults.

The relationship between self-rated health and willingness to vaccinate presents a notable paradox. Individuals who report better subjective health show lower willingness to vaccinate, potentially due to a diminished perception of risk. Conversely, vaccine hesitancy decreased within this group, indicating that those in better health, while feeling less compelled to seek immediate vaccination, do not harbor a strongly negative attitude toward vaccines in general. This paradox underscores an important opportunity for public health communication: addressing optimism bias by crafting targeted messages that effectively convey the preventive benefits of vaccination to older adults who perceive themselves as healthy. Furthermore, a history of chronic disease negatively impacts vaccination willingness; however, the overall effect remains slightly positive. This finding implies that while coexisting health conditions may discourage individuals from pursuing vaccination due to barriers such as inconvenient access to healthcare or low trust in vaccines, their heightened awareness of risk indirectly fosters vaccination behavior and mitigates hesitation. Future research should further explore the moderating roles of risk perception and health beliefs to elucidate this “health paradox.”

Socioeconomic status has a significant positive effect on vaccination, aligning with theories of health inequality [38]. This association may be partially explained by vaccine literacy. Previous studies have demonstrated that limited vaccine literacy significantly impedes older adults’ ability to process vaccination information and make informed vaccination decisions [29,39]. This finding highlights the necessity for policies that actively mitigate barriers encountered by low-SES groups. Such policies could include the expansion of free vaccination programs and the enhancement of health literacy initiatives to ensure equitable access to vaccines.

A comparison between the determinants of vaccination intention and uptake reveals critical insights into the decision-making process. Notably, a great effect observed for medical status in the uptake model disappeared in the intention model. For intention, chronic disease exerted a consistent positive influence through both direct and indirect pathways. This confirms that older adults with comorbidities possess a strong psychological willingness to vaccinate—likely driven by their crystallized health literacy and self-protection motivation [40,41]. The stark contrast between this high intention and the negative direct effect on actual uptake highlights a profound “Intention-Behavior Gap.” It suggests that while high-risk older adults want to get vaccinated, they are thwarted at the “last mile” of execution, likely by the aforementioned external barriers or misconceptions regarding contraindications. Furthermore, unlike the uptake model, socioeconomic status and subjective health exhibited “full mediation” patterns for intention. These factors did not directly drive willingness; rather, their influence was transmitted exclusively through reduced vaccine hesitancy [42]. This implies that for forming future intentions, the psychological battle is paramount: without addressing the core “trust deficit”, simply having higher socioeconomic status or health awareness is insufficient to generate willingness.

## 5. Limitations

Several limitations should be noted. First, this is a cross-sectional study, so we cannot establish causality. Second, participants were recruited from CHSCs, which may introduce selection bias, as these individuals are already visiting doctors and might care more about their health. Third, we only focused only on urban older adults and did not include rural populations. Finally, data on vaccination were self-reported.

## 6. Conclusions

This large-scale, multi-city study demonstrates that vaccine hesitancy is a central psychological barrier to influenza vaccination among older adults in urban China and that it mediates the effects of key sociodemographic and health-related factors on vaccination behavior. Physician recommendation emerged as the most powerful driver of uptake, with nearly half of its substantial total effect operating through the reduction in vaccine hesitancy—highlighting the irreplaceable role of primary care providers as trusted communicators and vaccine advocates.

## Figures and Tables

**Table 1:** Standardized Factor Loadings of Latent Constructs (CFA Results).

Latent Variable	Indicator	Std. All	*p*-Value
Vaccine Hesitancy	Confidence	0.769	<0.001
	Constraint	0.640	<0.001
	Calculation	0.408	<0.001
	Complacency	0.581	<0.001
	Collective Responsibility	0.737	<0.001
Medical History	Hypertension	0.492	<0.001
	Diabetes	0.510	<0.001
	Hyperlipidemia	0.513	<0.001
	Cerebrovascular Diseases	0.555	<0.001
	Other Cardiovascular Diseases Apart From Hypertension	0.478	<0.001
	Kidney Disease	0.477	<0.001
Socioeconomic Status	Education Level	0.827	<0.001
	Monthly Income	0.852	<0.001
Subjective Health	Health Status	0.689	<0.001
	Self-Care Capacity	0.519	<0.001

**Table 2:** Indexes of model fitness for the structural equation model.

Model	χ^2^/df	CFI	TLI	RMSEA	SRMR	PNFI
Vaccination Uptake	47.686	0.908	0.898	0.059	0.071	0.823
Vaccination Intention	46.594	0.916	0.908	0.058	0.070	0.831
Reference value	<5.000	>0.900	>0.900	<0.080	<0.080	>0.500

**Table 3:** Path diagram of the structural equation model for Vaccination Uptake.

	Estimate	S.E.	*p*-Value	Std. Est.
Vaccination uptake ← Vaccine hesitancy	−0.290	0.009	<0.001	−0.488
Vaccination uptake ← Subjective health	−0.433	0.146	0.003	−0.234
Vaccination uptake ← Medical status	−0.331	0.158	0.036	−0.152
Vaccination uptake ← Physician recommend	0.407	0.025	<0.001	0.191
Vaccination uptake ← Socioeconomic status	0.161	0.038	<0.001	0.125
Vaccine hesitancy ← Subjective health	−1.396	0.295	<0.001	−0.447
Vaccine hesitancy ← Medical history	−1.221	0.313	<0.001	−0.334
Vaccine hesitancy ← Physician recommend	−1.175	0.035	<0.001	−0.327
Vaccine hesitancy ← Socioeconomic status	−0.188	0.076	0.013	−0.087

**Table 4:** Effect of variables on the actual vaccination uptake.

Variable	Direct Effect	Indirect Effect	Total Effect
Physician recommend	0.191	0.160	0.351
Socioeconomic status	0.125	0.042	0.167
Medical status	−0.152	0.163	0.011
Subjective health	−0.234	0.218	−0.016

**Table 5:** Path diagram of the structural equation model for Vaccination Intention.

	Estimate	S.E.	*p*-Value	Std. Est.
Vaccination intention ← Vaccine hesitancy	−0.353	0.008	<0.001	−0.605
Vaccination intention ← Subjective health	0.193	0.129	0.137	0.105
Vaccination intention ← Medical history	0.422	0.144	0.003	0.197
Vaccination intention ← Physician recommend	0.274	0.023	<0.001	0.129
Vaccination intention ← Socioeconomic status	0.034	0.034	0.313	0.027
Vaccine hesitancy ← Subjective health	−1.433	0.304	<0.001	−0.453
Vaccine hesitancy ← Medical history	−1.253	0.320	<0.001	−0.340
Vaccine hesitancy ← Physician recommend	−1.181	0.035	<0.001	−0.325
Vaccine hesitancy ← Socioeconomic status	−0.181	0.078	0.020	−0.083

**Table 6:** Effect of variables on the actual vaccination intention.

Variable	Direct Effect	Indirect Effect	Total Effect
Physician recommend	0.129	0.197	0.326
Socioeconomic status	0.027 (ns)	0.050	0.077
Medical history	0.197	0.206	0.403
Subjective health	0.105 (ns)	0.274	0.379

Note: ns = not significant (*p* > 0.05).

## Data Availability

The data within this article will be shared upon reasonable request to the corresponding author.
